# Thermodynamic Simulation Calculations of Phase Transformations in Low-Aluminum Zn-Al-Mg Coatings

**DOI:** 10.3390/ma17112719

**Published:** 2024-06-03

**Authors:** Ziyue Zhang, Jie Zhang, Xingyuan Zhao, Xuequn Cheng, Xin Liu, Qifu Zhang

**Affiliations:** 1 Corrosion & Protection Center, University of Science & Technology Beijing, Beijing 100083, Chinachengxuequn@ustb.edu.cn (X.C.); 2 National Engineering Laboratory of Advanced Coating Technology for Metals, Central Iron & Steel Research Institute, Beijing 100081, China; cisrizhaoxy@126.com (X.Z.); 15810531277@163.com (X.L.);

**Keywords:** low-aluminum Zn-Al-Mg coatings, thermodynamic simulation, CALPHAD, MgZn_2_, Mg_2_Zn_11_

## Abstract

This study delves into the formation, transformation, and impact on coating performance of MgZn_2_ and Mg_2_Zn_11_ phases in low-aluminum Zn-Al-Mg alloy coatings, combining thermodynamic simulation calculations with experimental verification methods. A thermodynamic database for the Zn-Al-Mg ternary system was established using the CALPHAD method, and this alloy’s non-equilibrium solidification process was simulated using the Scheil model to predict phase compositions under varying cooling rates and coating thicknesses. The simulation results suggest that the Mg_2_Zn_11_ phase might predominate in coatings under simulated production-line conditions. However, experimental results characterized using XRD phase analysis show that the MgZn_2_ phase is the main phase existing in actual coatings, highlighting the complexity of the non-equilibrium solidification process and the decisive effect of experimental conditions on the final phase composition. Further experiments confirmed that cooling rate and coating thickness significantly influence phase composition, with faster cooling and thinner coatings favoring the formation of the metastable phase MgZn_2_.

## 1. Introduction

Over the past few decades, zinc–aluminum–magnesium (Zn-Al-Mg) coatings have garnered widespread attention due to their exceptional corrosion resistance, becoming an effective means of protecting steel structures from corrosion.

N. LeBozec et al. compared the corrosion performance of Zn-Al-Mg (Zn-Mg (1–2%)-Al (1–2%)) coatings in three accelerated corrosion tests commonly used in the automotive industry (VDA621-415, N-VDA, and Volvo STD 423-0014) with zinc–iron alloy (galvannealed, GA), zinc–aluminum (Zn-5Al, Galfan), traditional hot-dip-galvanized (HDG), and electrogalvanized (EG) coatings. The results showed that Zn-Al-Mg coatings performed better in open environments, especially in high salt load tests (e.g., VDA621-415) [1]. They further investigated the corrosion behavior of Zn-Al-Mg-coated steel in a marine environment. The results demonstrated that the microstructure with eutectic phases was the reason for the improved corrosion resistance of the coating [2]. Jae-Won Lee et al. investigated the cut-edge corrosion behaviors of Zn-Al-Mg-coated steel sheets in chloride-containing environments. Samples with a Zn-MgZn_2_ eutectic structure were fabricated via hot pressing and studied using electrochemical methods like the scanning vibrating electrode technique and conducting galvanic corrosion measurements. The results showed that the MgZn_2_ phase dissolved preferentially, promoting the co-precipitation of Mg(OH)_2_, Zn_5_(CO_3_)_2_(OH)_6_, and Zn_5_(OH)_8_Cl_2_·H_2_O, providing a longer-lasting corrosion inhibition mechanism [3]. Guang-rui Jiang et al. investigated the effects of different solidification processes on the microstructure and corrosion resistance of a Zn-Al-Mg alloy. The Zn-Al-Mg cast alloy was prepared using three different solidification processes: a water quench, air cooling, and furnace cooling. The microstructure was characterized using scanning electron microscopy (SEM). The results showed that by increasing the solidification rate, more aluminum remained in the primary crystals. Electrochemical analysis indicated that with a decreasing solidification rate, the corrosion current density of the Zn-Al-Mg alloy decreased, indicating higher corrosion resistance [4].

These alloy coatings not only have broad application prospects in fields such as automotive manufacturing, construction, and marine engineering but also continue to inspire researchers to delve into their fundamental properties and application potential [1,5,6]. Despite the excellent performance these coatings have demonstrated, the impact of their internal phase composition and phase transformation processes on the final performance of the coating remains a key topic of current research [7,8,9,10,11].

Previous studies have focused on the MgZn_2_ phase within Zn-Al-Mg coatings, considered a crucial factor in enhancing these coatings’ corrosion resistance [3,12,13]. However, as research progresses, some studies have also identified the presence of the Mg_2_Zn_11_ phase, indicating that the phase composition of these coatings is more complex than expected [8,14,15,16,17]. These findings have sparked the need for a deeper exploration of the phase transformation mechanisms occurring within these coatings, especially the transformation from MgZn_2_ to Mg_2_Zn_11_ and its specific impact on coating performance. Jaenam Kim et al. [7] conducted a detailed study on the structure and stoichiometry of Mg_x_Zn_y_ phases in Zn-Mg-Al hot-dipped coatings, particularly focusing on the formation of Mg_2_Zn_11_ and MgZn_2_ phases. Utilizing techniques such as X-ray diffraction (XRD) and Transmission Electron Microscopy (TEM), their study reveals that the addition of elements significantly impacts the fractions of these phases, while the Mg/Al ratio has a minor effect. Additionally, their research employed the CALPHAD method and thermodynamic databases to analyze the equilibrium and metastable states of these phases, demonstrating that the phase composition and microstructure of the coatings deviate from equilibrium under rapid solidification conditions. However, these authors did not conduct an experimental verification of the phase transformations in coatings under different process conditions, indicating that further research is still necessary.

In addition, a study by Minyun Xu et al. [15] highlights the importance of optimal hot-dip-coating process parameters for enhancing the crack resistance and corrosion resistance of Zn-Al-Mg coatings. Five different Zn-6Al-3Mg coatings were prepared using varying cooling rates: ZAM1 (0.1 °C/s), ZAM2 (1 °C/s), ZAM3 (5 °C/s), ZAM4 (30 °C/s), and ZAM5 (400 °C/s). The microstructures primarily consisted of an Al-rich phase, Zn/Al/MgZn_2_ or Zn/Al/MgZn_11_ ternary eutectics, and primary MgZn_2_ phase. However, unfortunately, there was no clear judgment or explanation of the formation law of the Mg_2_Zn_11_ and MgZn_2_ phases.

Based on the above research findings, there are still questions regarding the formation patterns of the microstructure in Zn-Al-Mg coatings. Furthermore, the phase transformation conditions within these coatings are not well understood and require further investigation. Given the time and cost limitations of many experimental methods, thermodynamic simulation calculations serve as an effective predictive tool, offering a new approach to understanding the phase behavior of Zn-Al-Mg alloy coatings. This study used thermodynamic simulations to predict phase composition and stability under various conditions, including the effects of alloy composition, temperature, and cooling rate. Subsequently, experiments were conducted to verify and analyze the simulation results, thus providing a clearer understanding of the phase transformation patterns in this coating.

## 2. Experimental Materials and Methods

### 2.1. Materials Preparation

**Figure 1:**
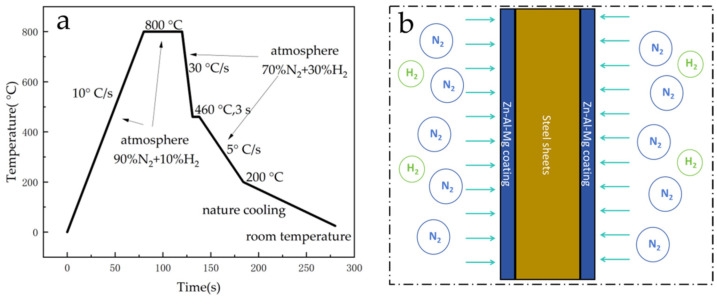
Experimental process control flow chart of hot-dip plating simulator. (**a**) Flowchart of the sample preparation process in which a simulator was used; (**b**) diagram of the post-coating cooling process.

The Zn-Al-Mg coating specimens were prepared using a self-developed hot-dip simulation machine (GCA-IV) in the laboratory. This machine can simulate hot-dip coating production lines under factory conditions, and conditions such as annealing temperature, immersion time, and post-coating cooling rate can be flexibly controlled. The equipment is connected through a transmission rod, driven by a motor that facilitates reciprocal vertical movement, enabling hot-dip coating experiments in various functional chambers. The steel substrate for the coating was a low-carbon interstitial-free (IF) cold-rolled thin plate produced industrially. The main route parameters for specimen preparation are shown in Figure 1a. The annealing temperature used was 800 °C. A zinc bath was kept at 450 °C until a steel plate was immersed in it at 460 °C for 3S. The coating thickness was kept at around 20 μm by adjusting the N_2_ gas knife flow rate. Finally, after being plated, the sample was cooled to room temperature at a rate of 5 °C/s. The protective atmosphere of steel plate annealing was 90% N_2_ + 10% H_2_, and the purging process of 70% N_2_ + 30% H_2_ was adopted in the cooling stage (Figure 1b), and the cooling rate was controlled by adjusting the atmosphere flow rate. Figure 1b illustrates a schematic of the cooling process after hot dipping, where the temperature is lowered by purging the chamber in a controlled atmosphere. The composition and identification numbers of the samples are shown in Table 1.

As the cooling process of the galvanizing simulator involves purging with a mixture of N_2_ and H_2_ gases, it cannot generate extremely rapid solidification conditions. Therefore, samples were prepared by rapidly cooling the molten alloy in a liquid medium. Zn-Al-Mg ingots created under varying cooling regimes were subjected to three different cooling methods: ambient air cooling, oil cooling, and water cooling. The alloy melt was heated to 460 °C in a crucible. At room temperature, aliquots of the molten alloy were dripped into containers filled with 500 mL of quench oil and distilled water, respectively, for instantaneous solidification before being sampled for analysis. The specimens for air cooling were allowed to cool passively in the atmosphere.

### 2.2. Characterization

The microstructures of the ZnAlMg coatings were investigated using a FEI Quant 650-FEG (Hillsboro, OR, USA) scanning electron microscope (SEM). The scanning acceleration voltage applied was 30 kV, and the working distance was 10 mm. The physical phases of the coatings were analyzed using a Bruker D8 ADVANCE X-ray diffractometer (XRD) (Billerica, MA, USA) with a Co target, a tube current of 40 mA, a tube voltage of 35 kV, and a scanning speed of 2°/min, along with a Lynxeye XE detector (Stockholm, Sweden).

## 3. Results and Analysis

### 3.1. Construction of the CALPHAD Theoretical Model

CALPHAD (Computational Phase Diagram Method) is a computational method used to predict the thermodynamic properties and phase equilibria of multi-component systems. Developed in the 1970s, this method combines thermodynamics with computational tools to accurately evaluate phase diagrams and other relevant thermodynamic data.

By researching existing binary system phase diagrams (including experimental and thermodynamic data), appropriate binary system phase diagrams and their thermodynamic data were assessed. Based on the thermodynamic data of existing binary systems, the CALPHAD method, combined with appropriate lattice models, was used to establish the thermodynamic expressions for all solid solutions, binary intermediate phases, and ternary phases, thereby constructing a thermodynamic database for the Zn-Al-Mg system.

**Figure 2:**
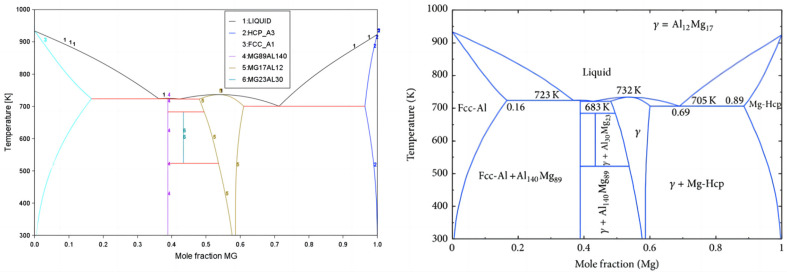
Mg-Al binary system. The left image shows the calculation results obtained in this work; the right image shows a phase diagram reported in the literature [18].

**Figure 3:**
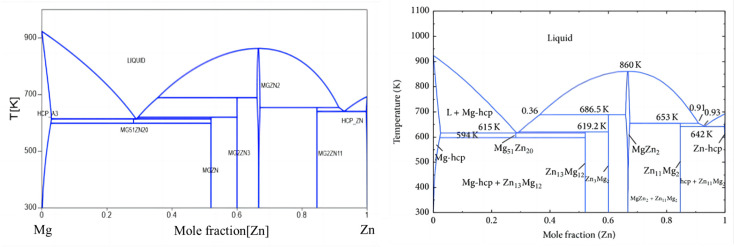
Mg-Zn binary system. The left image shows the calculation results obtained in this work; the right image shows the phase diagram reported in the literature [19].

**Figure 4:**
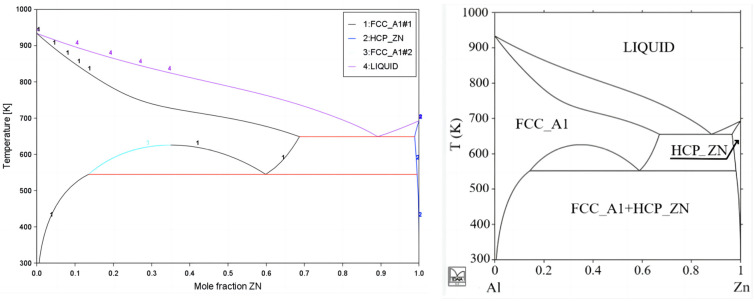
Al-Zn binary system. The left image shows the calculation results obtained in this work; the right image shows a phase diagram reported in the literature [20].

**Figure 5:**
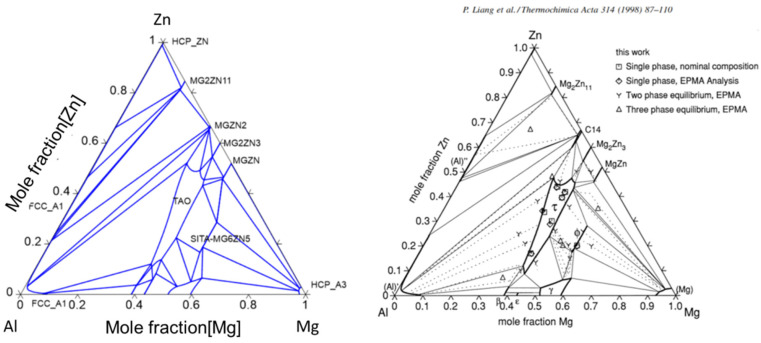
Isothermal section at 608 K of the Zn-Al-Mg ternary system. The left image shows the calculation results obtained in this work; the right image shows a phase diagram reported in the literature [21].

This work evaluated suitable binary system phase diagrams and their thermodynamic data by reviewing the literature on existing binary systems. Comparisons of the Zn-Al-Mg ternary system and its related binary systems, i.e., Mg-Al, Mg-Zn, and Zn-Al, are illustrated in Figure 2, Figure 3 and Figure 4. Figure 2 shows the evaluation of the Mg-Al binary system phase diagram, including phases such as Liquid, Fcc-Al, Hcp-Mg, Mg_89_Al_140_, Mg_23_Al_30_, and γ(Mg_17_Al_12_). Figure 3 is the evaluation of the Mg-Zn binary system phase diagram, including Liquid, Hcp-Zn, Hcp-Mg, Mg_51_Zn_20_, MgZn (Mg_12_Zn_13_), Mg_2_Zn_3_, MgZn_2_, and Mg_2_Zn_11_ phases. Figure 4 is an evaluation of the Al-Zn binary system phase diagram, including Liquid, Hcp-Zn, and Fcc-Al phases. Figure 5 compares the isothermal section at 608 K of the Zn-Al-Mg ternary system, including the aforementioned binary marginal phases, terminal phases, and TAO[τ-Mg_32_(Al,Zn)_49_] and SITA-Mg_6_Zn_5_[Φ-Mg_6_(Al,Zn)_5_]. By comparing these results with those reported in the literature, it was found that they are essentially consistent, indicating that the Zn-Al-Mg ternary system thermodynamic database constructed in this work is reasonable and reliable. Thus, it could be used to simulate the non-equilibrium solidification process in the next phase.

The calculation of solidification paths for binary alloys is relatively simple. This is because the phase diagram of a binary alloy corresponds to a two-dimensional plane, and the composition of the liquid phase changes strictly along the liquidus line, allowing the solidification path to be directly determined from the phase diagram.

However, the solidification path for ternary alloys is much more complex. The phase diagram expands from a surface to a volume, the liquidus line evolves into a liquidus surface, and the original eutectic point becomes a eutectic trough. Therefore, it is not possible to directly analyze the direction of solidification from a phase diagram. At this point, knowing the distribution of solutes is necessary to determine the solidification path. The phase diagram becomes more complex, and accordingly, the calculation of the solidification path becomes much more complicated. From this, it can be seen that the study of the solidification path of ternary alloys must be based on the micro solute redistribution model and phase diagram of the ternary alloy. On this basis, a reasonable algorithm can be established according to the composition, temperature, and phase diagram to determine the solidification path.

The solidification process can be determined by the composition and temperature within the volume elements of the solidification region based on the micro-segregation model and phase diagram, using a one-dimensional micro model to describe the process of solidification, and the relationship between solid/liquid phase composition and content changes during the solidification process. This calculation model is based on the Scheil model, considering the effect of compositional undercooling on the temperature field, as well as the effect of solute trapping in the rapid solidification process. A one-dimensional model was constructed using Matlab R2021b software, as shown in Figure 1b. The mathematical model is as follows:

Distribution of solutes in ternary alloy solidification (for two types of alloys, A and B):

Temperature field:

(1) $$\frac{\partial T}{\partial t}=D_{T}\nabla^{2}T+\frac{Lh}{c_{p}}\frac{\partial f}{\partial t}$$

*T* represents temperature, *t* represents time, *D_T_* represents thermal diffusivity, *L* represents diffusion depth, *h* is the heat transfer coefficient, *c_p_* is heat capacity, and *f* is the liquid phase fraction.

Solid–liquid phase fraction:

(2) $$f_{S}+f_{L}=1$$

$f_{S}$ represents the volume fraction of the solid phase, and $f_{L}$ represents the volume fraction of the liquid phase.

Concentration field:

(3) $$\frac{\partial {\left(\rho C_{n}\right)}_{m}}{\partial t}=\rho_{S}C_{nS}\frac{\partial f_{S}}{\partial t}+\int_{0}^{f_{s}}[\frac{\partial (\rho_{S}C_{nS})}{\partial t}]d\eta +\rho_{L}C_{nL}\frac{\partial f_{L}}{\partial t}+f_{L}\frac{\partial (\rho_{L}C_{nL})}{\partial t}$$

ρ represents density, and η represents concentration.

In the single-phase region,

(4) $$C_{Ln}=C_{0n}{\left[1-(1-\Phi_{n}^{\alpha }k_{n}^{\alpha })f_{s}\right]}^{\left(k_{n}^{\alpha }-1)/(1-\Phi_{n}^{\alpha }k_{n}^{\alpha }\right)}$$

(5) $$\Phi_{n}^{\alpha }=\theta_{n}^{\alpha }\cdot \varphi_{n}^{\alpha }/(1+\theta_{n}^{\alpha }\cdot \varphi_{n}^{\alpha })$$

$C_{Ln}$ represents the liquid phase composition of solute n (n = A, B), $C_{on}$ is the equilibrium partition coefficient in the component, $f_{S}$ represents the volume fraction of the solid phase, $f_{L}$ represents the volume fraction of the liquid phase, and α represents the solidifying phase. $\theta_{n}^{\alpha }$ refers to the solute diffusion parameters in the solid phase, while $\varphi_{n}^{\alpha }$ refers to the mass fraction of the solute on the solid phase side.

In the eutectic region,

(6) $$C_{Ln}^{2E}=C_{0n}{\left[1-(1-\Phi_{n}^{2E}k_{n}^{2E})f_{s}\right]}^{\left(k_{n}^{2E}-1)/(1-\Phi_{n}^{2E}k_{n}^{2E}\right)}$$

(7) $$\Phi_{n}^{2E}=\theta_{n}^{2E}\cdot \varphi_{n}^{2E}/(1+\theta_{n}^{2E}\cdot \varphi_{n}^{2E})$$

$C_{Ln}^{2E}$ represents the liquid phase composition of solute n (n = A, B), $C_{on}$ is the equilibrium partition coefficient in the component, $\Phi_{n}^{2E}$ is the comprehensive micro solid phase back-diffusion parameter of component n in the magnesium phase [0, 1], $k_{n}^{2E}$ is the equilibrium partition coefficient of solute n in the magnesium phase, $f_{S}$ represents the volume fraction of the solid phase, $\theta_{n}^{2E}$ refers to the solute diffusion parameters in the solid phase, and $\varphi_{n}^{2E}$ refers to the mass fraction of the solute on the solid phase side.

The eutectic solidification reaction of two phases is as follows: L → α + β.

Then, $k_{n}^{2E}$ is written as

(8) $$k_{n}^{2E}=g_{\alpha }^{2E}\cdot k_{n}^{\alpha }+g_{\beta }^{2E}\cdot k_{n}^{\beta }=g_{\alpha }^{2E*}\cdot k_{n}^{\alpha }+(1-g_{\alpha }^{2E*})\cdot k_{n}^{\beta }$$

$g_{\alpha }^{2E*}$ represents the mass fraction of the α phase in the solid at the solid/liquid interface.

$k_{n}^{\alpha }$ represents the equilibrium partition coefficient of component n for the α phase at the solid/liquid interface.

$k_{n}^{\beta }$ represents the equilibrium partition coefficient of component n for the β phase at the solid/liquid interface.

### 3.2. Simulation Calculation Results

**Figure 6:**
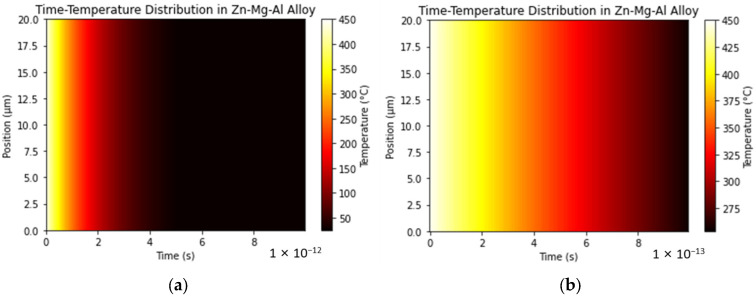
Zn-2Al-2Mg coating with a thickness of 20 μm, a solidification initiation temperature of 450 °C, and a boundary temperature of 25 °C; the temperature of the Zn-Mg-Al alloy changes over time along the thickness direction. (**a**) A two-dimensional heat map of the time range (0, 1 × 10^−12^ s). (**b**) A two-dimensional heat map of the time range (0, 1 × 10^−13^ s).

The simulation conditions were set under ideal circumstances, with a thermal conductivity of 150 W/mK, a density of 7000 kg/m^3^, and a specific heat capacity of 380 J/(kg·K). According to the simulation results (Figure 6), the temperature dropped from 450 °C to 250 °C in just 1 × 10^−13^ s. The initial estimate for the cooling rate was (450–250 °C)/1 × 10^−13^ s = 2 × 10^15^ °C/s, indicating a very rapid rate of temperature change, which has already reached the temperature change speed required for extremely rapid solidification.

The simulation conditions were set to match the parameters of the laboratory simulation machine, with a cooling rate of 5 °C/s. The proportions of MgZn_2_ and Mg_2_Zn_11_ in the ZAM1-3 coatings, as shown in Table 2, indicate that Mg_2_Zn_11_ is the dominant phase in the coatings at a cooling rate of 5 °C/s. The primary reason for this is the slow change in temperature, which allows the metastable phases formed first by the eutectic reaction (9) to transform into Mg_2_Zn_11_ through the peritectic reaction (10).

Liquid → η-Zn + α-Al + MgZn_2_ (9)

Liquid + MgZn_2_ → Mg_2_Zn_11_ (10)

**Figure 7:**
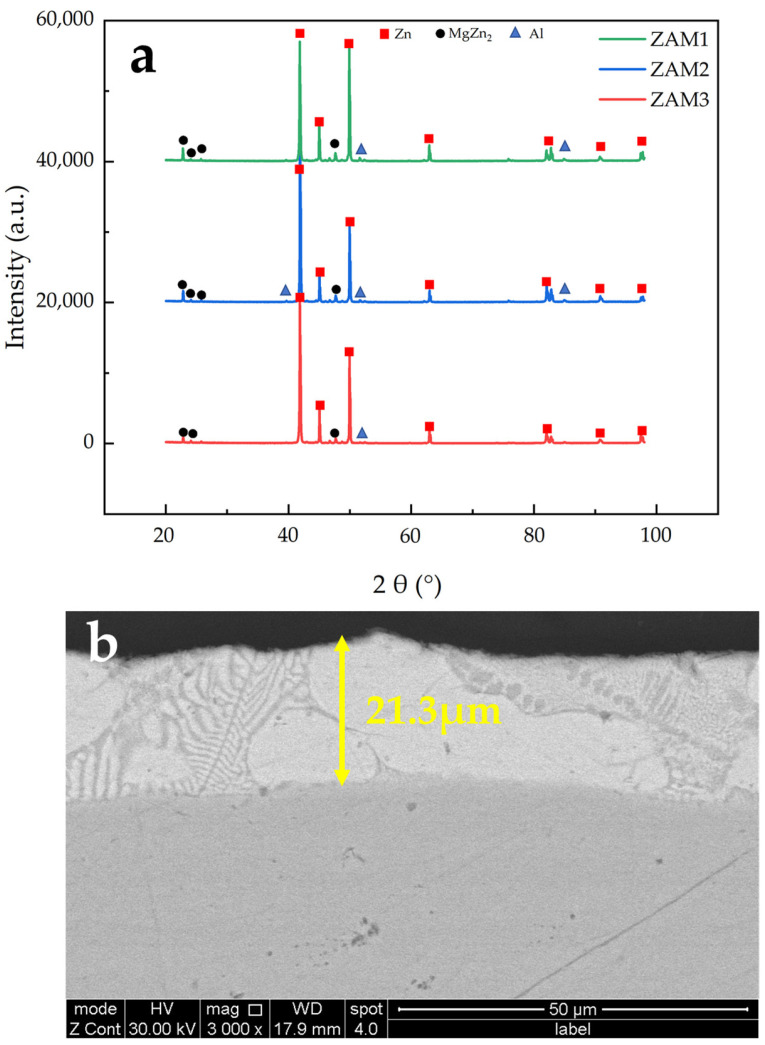
Experimental preparation of coatings: (**a**) XRD phase analysis; (**b**) cross-sectional thickness of the ZAM2 coating.

To verify the simulation results, samples of three different compositions were prepared using a hot-dip galvanizing simulator, and the coatings were analyzed using XRD phase analysis (Figure 7a). The experimentally prepared coatings did not exhibit the Mg_2_Zn_11_ phase, containing only Hcp-Zn, Fcc-Al, and MgZn_2_ phases. This indicates that there was no Mg_2_Zn_11_ in the sample coating, or its content was too low to be detected via XRD. This finding aligns with results reported in the literature [3], which indicate that the phases present in this coating include Hcp-Zn, Fcc-Al, and MgZn_2_. The paper specifically highlights the fact that MgZn_2_ plays a crucial role in endowing this coating with superior resistance to edge corrosion. The absence of the Mg_2_Zn_11_ phase in experimentally prepared coatings may be attributed to the cooling rate applied and coating thickness, as shown in Figure 7b. The thickness of the coating was maintained at approximately 20 microns via the action of the air knife in the simulator, resulting in a continuous and complete coating. Additionally, the morphological features of the coating’s cross-section are distinctly clear. Coating thickness can significantly impact the efficiency of heat transfer within a coating, which is vital for the cooling process post-coating. Consequently, further simulations were performed alongside comparative analyses of coating thickness and cooling rates against the experimental samples.

**Figure 8:**
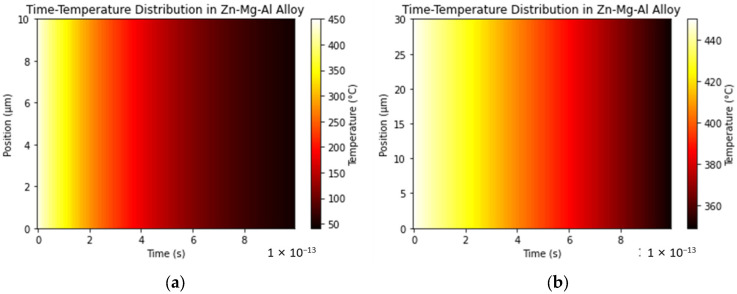
The temperature changes over time in the Zn-2Al-2Mg coating along the thickness direction at an initial temperature of 450 °C and a boundary temperature of 25 °C within a time range of (0, 1 × 10^−13^ s) in a two-dimensional heat map (**a**) for a coating thickness of 10 μm and (**b**) for a coating thickness of 30 μm.

By comparing Figure 6b with Figure 8a, it can be found that as the thickness decreases, the cooling rate becomes (450–50 °C)/1 × 10^−13^ s = 4 × 10^15^ °C/s for a 10 μm coating thickness and (450–350 °C)/1 × 10^−13^ s = 1 × 10^15^ °C/s for a 30 μm coating thickness. A faster cooling rate leads to an increase in the number of metastable phases. In the phase diagram of the Zn-Mg-Al alloy, it can be seen that through the eutectic reaction (13), the ternary eutectic phase of η-Zn/α-Al/MgZn_2_ is formed, while there is not enough time for the stable phase Mg_2_Zn_11_ to form.

**Figure 9:**
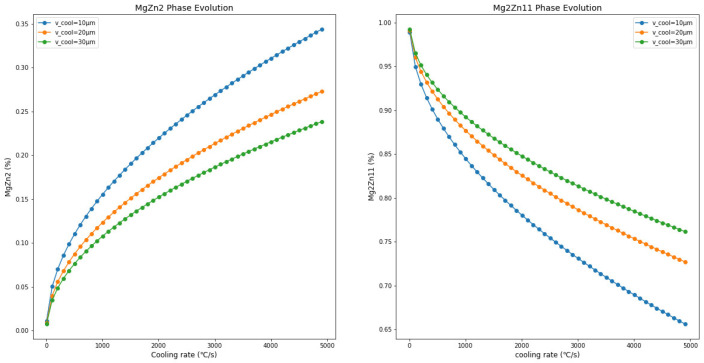
The trends of changes in the composition of the Zn-1Al-1Mg coating with variations in thickness and cooling rate.

**Figure 10:**
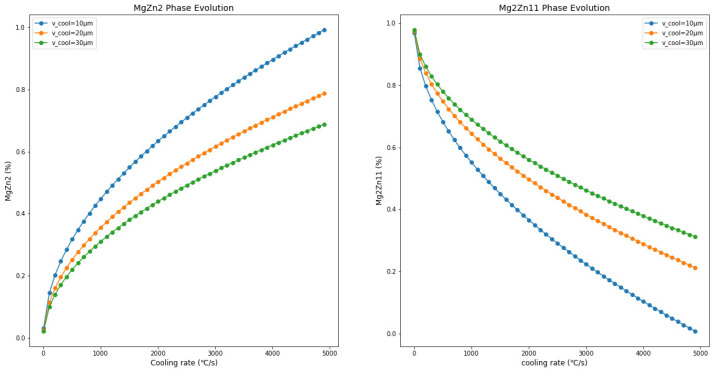
The trends of changes in the composition of the Zn-2Al-2Mg coating with variations in thickness and cooling rate.

**Figure 11:**
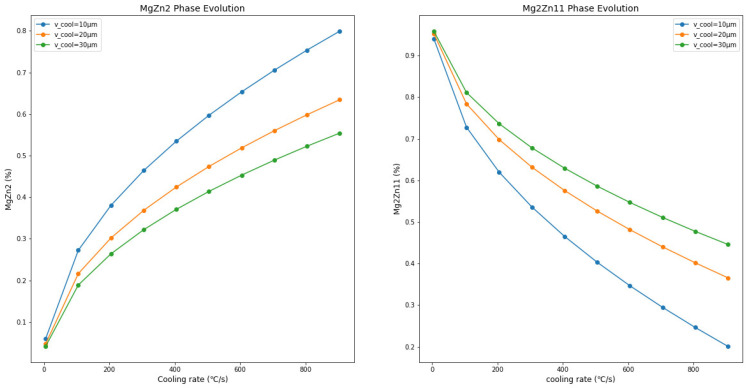
The trends of changes in the composition of the Zn-3Al-3Mg coating with variations in thickness and cooling rate.

As shown in Figure 9, reducing the thickness of the Zn-Mg-Al alloy coating leads to an increase in the metastable MgZn_2_ phase content and a decrease in Mg_2_Zn_11_ phase content. Comparisons with Figure 9, Figure 10 and Figure 11 reveal that, with the increase in Mg and Al content (Zn-1Mg-1Al, Zn-2Mg-2Al, Zn-3Mg-3Al), with the same coating thickness and cooling rate conditions, the proportion of MgZn_2_ formed in the coating increases. For example, at a cooling rate of 800 °C/s and a coating thickness of 30 µm, MgZn_2_ accounts for 0.08% in Zn-1Mg-1Al, 0.22% in Zn-2Mg-2Al, and 0.51% in Zn-3Mg-3Al. Meanwhile, the proportion of Mg_2_Zn_11_ correspondingly decreases. This indicates that increasing the Mg and Al content in the coating promotes the formation of MgZn_2_. When the cooling rate is 800 °C/s and the coating thickness is 10 µm, MgZn_2_ accounts for 0.13% in Zn-1Mg-1Al, 0.40% in Zn-2Mg-2Al, and 0.74% in Zn-3Mg-3Al. This shows that a thinner coating is more conducive to the formation of MgZn_2_.

### 3.3. The Impact of Cooling Rate and Thickness on Coatings

**Figure 12:**
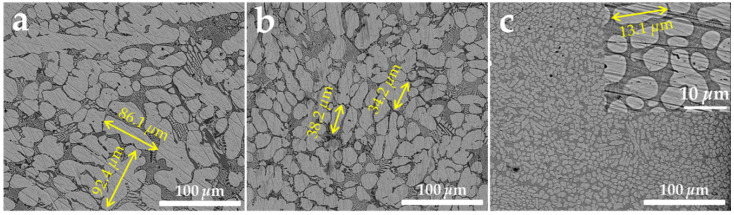
SEM surface images of Zn-3Al-3Mg alloy using different cooling techniques and rates: (**a**) water cooling, (**b**) oil cooling, and (**c**) air cooling.

To verify the results of the simulation calculations, the samples were prepared in the laboratory using a liquid alloy with a composition of Zn-3Al-3Mg cooled using three different techniques and rates: air cooling (40 °C/s), oil cooling (200 °C/s), and water cooling (600 °C/s). Small volumes of the alloy droplets were cooled using these three methods. SEM micrographs (as shown in Figure 12) clearly reveal significant differences in the microstructures of the alloy ingots as a result of changes in cooling rates. With an increase in cooling speed, the grain size of the coating becomes finer. When air-cooled, the maximum size of the zinc-rich phase in the ingots can exceed 90 μm; when oil-cooled, it is around 30 μm; and when water-cooled, the grain size of the ingots is less than 15 μm, with a more uniform distribution.

**Figure 13:**
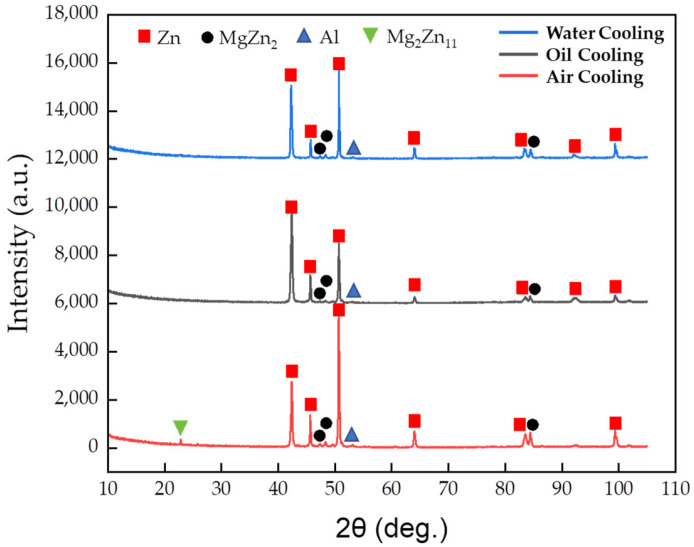
XRD phase analysis results of Zn-3Al-3Mg alloy cooled at different rates.

The XRD results (Figure 13) show that Mg_2_Zn_11_ diffraction peaks were found only in the ingots cooled under air conditions, while these peaks were not detected in the other two types of ingots. Instead, only the Hcp-Zn, Fcc-Al, and MgZn_2_ phases, similar to the coating’s XRD results (Figure 7a), were observed. This is consistent with our simulation calculations, indicating that the slower the cooling rate, the higher the content of the stable phase Mg_2_Zn_11_. The absence of Mg_2_Zn_11_ in the results relating to water cooling and oil cooling might be due to its low content or the presence of only the metastable phase MgZn_2_. Under conditions of faster cooling rates, MgZn_2_ is more likely to be present in the coating.

## 4. Conclusions

Thermodynamic simulation calculations were made using the CALPHAD method, and phase result verification analysis of hot-dip galvanized steel sheets prepared using a galvanizing simulator was performed. This paper analyzes the reasons for the phase transformation behavior in low-aluminum Zn-Al-Mg coatings and the methods for controlling it. By establishing a thermodynamic database for the Zn-Al-Mg ternary system and simulating the alloy’s non-equilibrium solidification process using the Scheil model, this study has unveiled the formation and transformation patterns of the MgZn_2_ and Mg_2_Zn_11_ phases within these coatings. These conclusions provide valuable theoretical foundations for guiding parameter optimization in the actual production process. Based on the key findings of this research, here is a summary of the main conclusions:

1. The dominant factors influencing the presence of Mg_2_Zn_11_ and MgZn_2_ phases in the coating: Although the initial simulation results suggested the potential predominance of the Mg_2_Zn_11_ phase under Scheil model conditions, experimental detection revealed the MgZn_2_ phase to be the main existing phase. This discrepancy indicates that under actual cooling conditions, the rapid cooling and micro segregation of alloy components might lead to the preferential formation of the MgZn_2_ phase, highlighting the complexity and dynamic nature of the non-equilibrium solidification process under experimental conditions.
2. The complex impact of cooling rate and coating thickness on phase composition: Variations in cooling rate and coating thickness significantly determine the proportions of the MgZn_2_ and Mg_2_Zn_11_ phases in the coating. Faster cooling rates and thinner coatings tend to promote the formation of the metastable MgZn_2_ phase, while slower cooling rates facilitate the stability of the Mg_2_Zn_11_ phase, as confirmed by experimentally prepared coating samples and XRD analysis. Higher cooling rates will promote the refinement of the coating’s microstructure.
3. Consistency and differences between experimental and simulation results: By comparing the experimental and simulation results, we recognize that although thermodynamic simulation provides valuable theoretical predictions, the failure to consider the effects of molecular dynamics resulted in a lack of understanding of the phase transformation principles of Mg_2_Zn_11_ and MgZn_2_ in the simulation results. This should be further explored in future research.

## Figures and Tables

**Table 1:** The compositions of experimental coatings.

Sample No.	Component	Mg (wt.%) (±0.1)	Al (wt.%) (±0.1)	Zn (wt.) (±0.1)
ZAM1	Zn-1Al-1Mg	1.1	1.2	97.7
ZAM2	Zn-2Al-2Mg	2.2	1.9	95.9
ZAM3	Zn-3Al-3Mg	3.2	3.1	93.7

**Table 2:** The proportions of MgZn_2_ and Mg_2_Zn_11_ in different coatings were determined through simulation calculations.

Sample No.	Component	Mg (wt.%) (±0.1)	Al (wt.%) (±0.1)	Zn (wt.%) (±0.1)	MgZn_2_ (wt.%)	Mg_2_Zn_11_ (wt.%)
ZAM1	Zn-1Al-1Mg	1	1	97.34	0.87	99.13
ZAM2	Zn-2Al-2Mg	2	2	97.16	2.51	97.49
ZAM3	Zn-3Al-3Mg	3	3	95.79	4.78	95.22

## Data Availability

The original contributions presented in the study are included in the article, further inquiries can be directed to the corresponding author.
